# Assessment of different organic supplements for degradation of *Parthenium hysterophorus* by vermitechnology

**DOI:** 10.1186/s40201-015-0203-1

**Published:** 2015-05-16

**Authors:** R Hiranmai Yadav

**Affiliations:** School of Natural Resources Management and Environmental Sciences, College of Agriculture and Environmental Sciences, Haramaya University, P.O. Box #337, Dire Dawa, Ethiopia

**Keywords:** *Eisenia fetida*, Field wastes, Nutrient content, Vermicompost, Weeds, Parthenium

## Abstract

Globally, there are a number of treatments indicated for the control of invasive alien plant species like parthenium. The production and use of vermicomposts from weeds or other wastes in agriculture is economical and eco-friendly. The unique advantage of using vermicomposting is that it helps to build and sustain soil condition and fertility for sustainable agricultural activities. The present study was an attempt to produce the vermicompost from Parthenium, farm and animal wastes and to analyze its nutrient content and suitability to be used as manure. The raw materials Parthenium, farm and animal wastes were collected and decomposed by tank method using *Eisenia foetida*. There were four different treatments in three replications of parthenium mixed with farm wastes and animal manures @10:1:1 ratio. The pH, organic carbon, organic matter, macro and micro nutrients and exchangeable bases were analyzed by standard methods. Addition of different farm and animal wastes helped to degrade the Parthenium and improve the nutrient value. Different treatments have shown improvements in the degraded product in terms of pH, organic carbon, organic matter, macro and micro nutrients and exchangeable bases. The vermicompost was found to have a good quality comparable to any organic manure. The results revealed the economic feasibility of the vermicompost (the organic manure) production as it uses the locally available materials and eco friendly nature of its technology. This methodology can be adopted by farmers to improve the crop productivity and maintain the soil fertility using the locally available organic waste materials.

## Introduction

Organic farming involves the use of eco friendly manures in agriculture. The increased use of chemical fertilizers improved the production but at same time the soil fertility is getting reduced due to inadequate organic matter. To combat this, the use of organic materials are recommended. The vermitechnology is a promising technique for recycling the weeds and wastes and the resultant product improves the soil fertility and crop production without harming the nature. It is easy to practice, ecologically safe and used to reduce the pollution problems. Different experiments are being carried out to emphasize the importance and utility of this technology. The practice of vermiculture is at least a century old and now it is receiving worldwide attention as a waste management technique in terms of weeds utilization and reduction in quantity of accumulated wastes. The resultant product of vermicomposting is a nutrient rich input for crop production [1].

Reduction of wastes by co composting using chicken slurry and pineapple wastes by [2] have been found to be a digestibility of solid waste and improved nutrient balance. The co composting by improved nutrient release reduces the cost and use of fertilizers. The cattle manure, food waste and paper vermicomposts was found to be rich in nutrients and vermicomposts contains plant growth regulating materials like plant growth hormones and humic acids that can increase the germination, growth and yield of plants [3, 4].

Studies by [5] have also reported that the macronutrients were improved by the decomposition of Lantana with animal manures and earthworms. Earlier studies by [6] showed that the decomposition of Lantana residue with oak pine leaf litter observed that there is high rate of nutrient release from the decomposing materials. Mixing of Lantana with other mulch materials positively influenced the decomposition and release of nutrients. Banta and Dev [7] have also reported low C/N ratio of the composts prepared from Lantana.

Earthworm excreta are excellent soil conditioning material with a high water holding capacity and a natural time release for releasing Nitrogen (N) into the soil [8]. The higher concentration of N in vermicompost coupled with its greater availability would be expected to make it a superior soil amendment for plant growth [9]. Total porosity and non-capillary porosity were improved in soil due to municipal compost application [10]. The highest reduction in percentage of phenols was observed in *Parthenium* compost. *Parthenium* recorded higher content of phenols in fresh forms [11]. Suthar and Sharma [12] have vermicomposted Lantana with cowdung using *Eisenia foetida* and recorded a decrease in pH, total organic carbon and C/N ratio while there was an increase in ash content, total nitrogen, available phosphorus, exchangeable potassium and calcium and nitrate nitrogen.

*Parthenium hysterophorus* commonly called as congress grass or carrot grass is a poisonous, allergic and obnoxious weed of fields, barren land and forest areas [13]. Since its introduction the weed is reported as relentlessly spreading throughout the agricultural lands, forests, orchards, poorly managed arable crop lands and rangelands almost throughout the country [14]. The Parthenium weed infestation increases the total stand density and biomass. It has the ability to utilize the drought prone area that leads to the deterioration of indigenous species and leave much bare ground cover [15].

The invasion of Parthenium to agricultural land is a serious problem and there is no practically effective weeding method for its eradication or control. It is increasing every year and the removal or elimination of the plant is becoming more complicating. The studies by have also shown that *Parthenium hysterophorus* can be a raw material for vermicomposting when mixed with cow dung and pressmud in equal proportion [16]. Integrated efforts should be made to suppress and get rid of the weed. The farm wastes and animal wastes of diverse types are available in field after harvest. These nitrogen rich materials could be used as an additive to enhance the decomposition of weeds like Parthenium. The wastes those are available in the farm when added to the vermicomposting process that will enhance the degradation and enrich the nutrient value of the resultant product. The present work was an attempt to decompose Parthenium with farm wastes and different animal manures and assess its manurial value.

## Methods

A study was carried out to assess the impact of application of farm wastes and animal manures in decomposing the parthenium.

### Substrates and combinations used in compost preparation

The materials were collected within the campus field and animal farms. There were four different treatments of parthenium mixed with farm wastes and animal manures @10:1:1 ratio. The nutrient content of fresh Parthenium is expressed in Table 1.

**Table 1:** Nutrient status of fresh Parthenium

S No	Nutrient content	Quantity
1.	Organic carbon (%)	2.02
2.	Organic matter (%)	3.49
3.	Nitrogen (%)	0.62
4.	Phosphorus (%)	0.10
5.	Potassium (%)	0.32
6.	Zinc (ppm)	178.00
7.	Copper (ppm)	76.00
8.	Iron (ppm)	84.00
9.	Manganese (ppm)	125.00
10.	C:N ratio	3.26
11.	C: P ratio	20.20

Source: PhD thesis, R. Hiranmai Yadav

- *T*_*1*_*- Parthenium hysterophorus* plants + farm wastes + Cow dung
- *T*_*2*_*- Parthenium hysterophorus* plants + farm wastes + Goat manure
- *T*_*3*_*- Parthenium hysterophorus* plants + farm wastes + Poultry manure
- *T*_*4*_*- Parthenium hysterophorus* plants + farm wastes + Swine manure

### Vermicomposting process

The process was carried out in cement tanks of 1 m × 1 m × 1 m size. The cement tanks were constructed under shade. The compost unit is constructed with permanent materials for continuous process. The Parthenium plants were collected before flowering stage and chopped into smaller pieces of 1–2 in. mechanically and decomposed with farm wastes and animal wastes for about 20 days. This was to reduce the size of the particles that can facilitate the feeding of earthworms. The earthworm *Eisenia fetida* was used for the decomposition process. The climatic conditions were favorable and temperature and moisture were maintained by sprinkling water regularly. After 20 days the materials were mixed and temperature was checked to release the earthworms. About 1Kg earthworms were released into each tank. After release of worms to the tank, precautions were taken to protect the worms from predators. The vermicomposting process was carried out for duration of 60 days.

### Analysis of physicochemical parameters

The nutrient contents were analysed by standard procedures. The vermicompost was collected and analysed for different parameters to assess the nutrient status.

#### pH

pH was measured using digital pH meter.

#### Estimation of organic carbon and organic matter

The Organic carbon (C) content was estimated following [17] and organic matter was calculated from it. For the analysis 0.5 g of soil was weighed (passed through 0.2 mm sieve) and transferred it to a 250 ml/500 ml conical flask. 10 ml of 1 NK_2_Cr_2_O_7_ was added and mixed well by swirling the flask. Then 20 ml of con. H_2_SO_4_ was added and mixed by gentle rotation for one minute to ensure complete contact of the reagent with the soil. Allowed the contents to stand for 20–30 min. The flask was kept preferably on an asbestos sheet to avoid burning of table due to intense heat. The reaction mixture was diluted with 200 ml distilled water. 10 ml of phosphoric acid and 1 ml of diphenylamine indicator were added. Titrated the solution with 0.5 N ferrous ammonium sulphate. The colour was dull green at the beginning and then shifted to a turbid blue as the titration proceeded. The end point was very sharp. At the end point the colour sharply shifted to a brilliant bright green. The organic carbon content in the sample was determined using the formula,$\mathrm{Per}\ \mathrm{cent}\ \mathrm{organic}\ \mathrm{carbon}=\frac{\left(\mathrm{B}.\mathrm{V}.-\mathrm{S}.\mathrm{V}.\right)\times 10\times 0.003\times 100}{\mathrm{B}.\mathrm{V}.\times \mathrm{W}}$

Organic matter was calculated by multiplying the value of organic carbon by a standard value of 1.724.

#### Estimation of total Nitrogen (N)

The macro nutrients namely total Nitrogen (N) was calculated by Micro kjeldahl method [18]. One g of sample was transferred into a 100 ml conical flask. 15 ml of diacid mixture (sulphuric acid and perchloric acid in the ratio of 5: 2) was added and covered the mouth of the flask with a funnel. Digested the contents of the flask over a sand bath till a clear solution was obtained. The contents of the flask were transferred into a 250 ml volumetric flask using minimum quantity of water and made up the volume to 250 ml.10 ml of the diacid extract was pipetted out into a distillation flask. 25 ml of 2 % boric acid was pipetted out into an ice tumbler/beaker and two drops of double indicator (bromocresol green methyl red) was added and kept it at the delivery end of the distillation set.10 ml of 40 % sodium hydroxide was added to the distillation flask and distilled the contents and the distillate was collected in 2 % boric acid. Tested the completion of distillation with a moistened red litmus paper. Absence of blue colour indicated that all ammonia had been distilled. The delivery tube was washed with distilled water and the washings were collected in ice tumbler. Titrated the contents with N/10 sulphuric acid. The end point was the change of colour from green to pinkish.

#### Calculation

Weight of the sample taken = 1 g

Volume of diacid extract prepared = 100 ml

Volume of diacid extract pipetted out for analysis = 10 ml

Volume of N/10 H_2_SO_4_ consumed = X ml

1 ml of N/10 H_2_SO_4_ = 0.0014 g of N

This is present in 10 ml of the diacid extract

Therefore in $$ 250\ \mathrm{ml} = \frac{\mathrm{X}\times 0.0014\times 100}{10}\times \mathrm{g}\kern0.5em \mathrm{of}\kern0.5em \mathrm{N} $$

This is present in 1.0 g of the sample in $100\ \mathrm{g} = \frac{\mathrm{X}\times 0.0014\times 100\times 100}{10}\times \mathrm{g}\kern0.5em \mathrm{of}\kern0.5em \mathrm{N}$

% of nitrogen on moisture free basis = $\frac{\mathrm{X}\times 0.0014\times 100\times 100}{10}\times \frac{100}{\left(100-\mathrm{M}\right)}$ (M = Moisture content of the sample)

#### Estimation of Phosphorus (P) and micronutrients

The amount of phosphorus and micronutrients in the sample was estimated by the method of [19].

#### Estimation of phosphorus (P)

5 ml of the triple acid extract was pipetted out into a 25 ml volumetric flask. 5 ml of Barton’s reagent was added, shaken well and made up the volume to 25 ml and made it a homogenous solution. Allowed to stand for about 30 min. for the colour to develop. The intensity of yellow colour developed was read in a spectrophotometer at 470 nm. By referring to the standard curve prepared calculated the phosphorus content of the sample.

#### Calculation

Weight of sample taken = w g/g

Volume of triple acid extract prepared = V ml

Aliquot taken for colour development = 5 ml

Concentration of the solution as read from the spectrophotometer = x ppm

Therefore P content on moisture free basis = $\frac{\mathrm{x}}{10^6}\times \frac{25}{5}\times \mathrm{V}\times \frac{100}{1\mathrm{w}}\times \frac{100}{\left(100-\mathrm{M}\right)}$ (M – Moisture content of the sample)

#### Estimation of micronutrients

The micronutrients like Zinc (Zn), Copper (Cu), Iron (Fe) and Manganese (Mn) were also estimated. From the working standard solutions, the standard curve was prepared for each nutrient. In between two standard solutions, introduced the blank and ensured that there is no change in the zero point. Prepared a graph by plotting the absorbance values against concentrations. Transferred 10 g of air dried soil sample to 150 ml conical flask/polythene bottle and added 20 ml of the DTPA extractant solution. Closed the bottle and shaked for 2 h in a horizontal shaker. Filtered through whatman No. 42 filter paper. Take a reading of the filtrate in the atomic absorption spectrophotometer and by referring to the standard curve calculate the concentration of each micronutrient in the sample. Shaking time, concentration and pH of the DTPA extractant and temperature will influence the quantity of nutrients extracted. The exchangeable bases Sodium (Na) by 1 M ammonium acetate method, Calcium (Ca), Magnesium (Mg) [20] and Potassium (K) [21] contents were also analysed. C: N and C: P ratios were calculated.

## Results and discussion

**Fig. 1:**
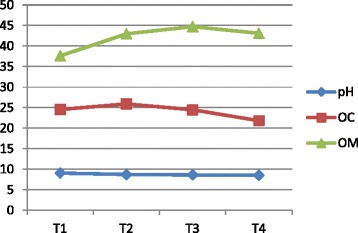
pH, Organic carbon (%) and Organic matter content (%) of different vermicomposts

**Fig. 2:**
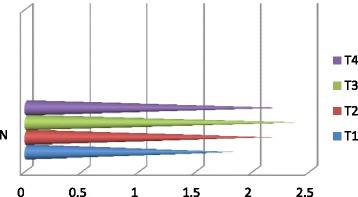
Nitrogen (%) content of different vermicomposts

**Fig. 3:**
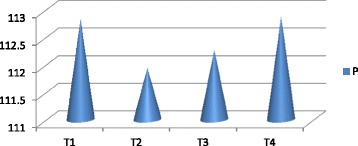
Phosphorus content of different vermicomposts

**Fig. 4:**
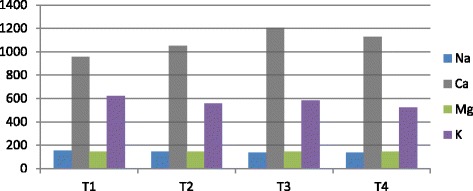
Exchangeable bases of different vermicomposts

**Fig. 5:**
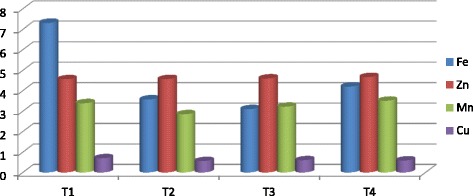
Micro nutrients contents of different vermicomposts

**Fig. 6:**
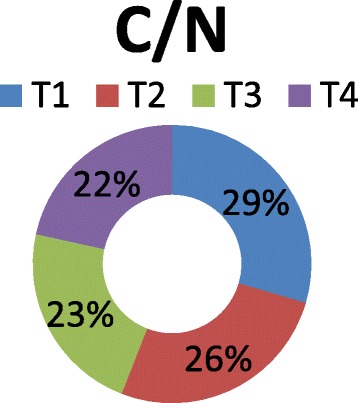
C/N ratio of different vermicomposts

**Fig. 7:**
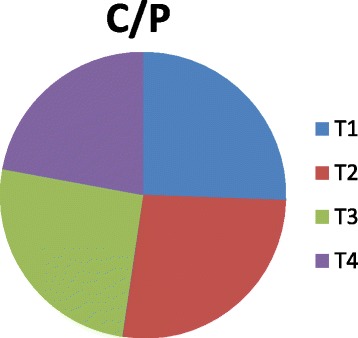
C/P ratio of different vermicomposts

The vermicompost harvested after sixty days of decomposition was dark brown in color and homogeneous in nature. The product had an earthy aroma characteristic of the matured compost. The biomass of earthworms was also increased considerably from the initial added number. It indicates that the substrates were suitable for the multiplication of the organisms. Growth and reproduction of worms require sufficient nutrient from the substrates. The palatability of the substrate materials are of significance for multiplication and thereby conversion into a nutrient rich organic manure by the earthworm activity. The decomposition of the organic wastes improves the nutrient value of the end products (Table 2). The pH was alkaline in all the vermicomposted samples that ranged from 9.09 in Parthenium vermicomposted with farm wastes and cow dung (T_1_) to 8.52 in T_4._ The organic carbon and organic matter varied considerably among the different treatments (Fig. 1). The nitrogen and phosphorus contents were also found to be significant in different treatments (Figs. 2 and 3). The exchangeable bases are depicted in Fig. 4 and micro nutrients in Fig. 5. The exchangeable bases potassium, sodium and magnesium and micro nutrients copper and iron were also found to be high in T_1_. The calcium content was higher in T_3_ whereas the zinc and manganese were in T_4_. The C/N and C/P ratios are represented in Figs. 6 and 7. They were within the recommended range for organic manures. All the nutrient contents were improved by the treatments with different organic supplements.

**Table 2:** Nutrient status of Parthenium vermicomposted with farm and animal wastes

S No	Parameters	T1	T2	T3	T4
1.	pH	9.08	8.72	8.63	8.52
2.	OC(%)	24.56	25.90	24.48	21.83
3.	OM(%)	37.61	42.96	44.73	43.12
4.	N(%)	1.83	2.16	2.39	2.23
5.	P(ppm)	112.83	111.94	112.27	112.87
6.	K(ppm)	619.88	555.58	581.63	522.46
7.	Na(ppm)	154.58	145.40	133.92	137.25
8.	Ca(ppm)	955.94	1050.70	1204.98	1127.52
9.	Mg(ppm)	146.25	144.23	143.40	145.30
10.	Cu(ppm)	0.70	0.56	0.60	0.59
11.	Fe(ppm)	7.31	3.57	3.10	4.20
12.	Zn(ppm)	4.56	4.57	4.6	4.67
13.	Mn(ppm)	3.39	2.85	3.22	3.51
14.	C/N	13.42	11.99	10.24	9.79
15.	C/P	0.22	0.23	0.22	0.19

Similar observations are made by [22] in decomposition of Parthenium with *Eisenia foetida*. They reported that utilization of organic supplements for the degradation of Parthenium helps to improve the nutrient value in addition to the reduction of weed biomass. The resultant product could be a good source of organic manure.

The earlier studies also showed that the pH of the fresh Parthenium was slightly acidic (6.84) whereas it turned alkaline in composted (8.89) and vermicomposted (8.53) samples. The content of organic carbon (C) and organic matter varied among the fresh and decomposed samples. It was reduced in composted and vermicomposted Parthenium. The fresh Parthenium recorded the lowest per cent of total Nitrogen (N), Phosphorus (P) and Potassium (K) compared to decomposed materials. Zinc (Zn) and Copper (Cu) contents were lower in fresh Parthenium compared to the composted and vermicomposted Parthenium. The Iron (Fe) content was lower in composted Parthenium compared to both fresh and vermicomposted samples. Manganese (Mn) content was observed to be low in fresh, than composted and vermicomposted Parthenium [23].

A tremendous increase in organic carbon and potassium content of vermicompost prepared from farm and weed wastes (papaya leaf litter, glyricidia loppings and pigeon pea stalks) was recorded [24]. The application of this compost increased the nitrogen, potassium and organic carbon content of the soil and thereby improving the congenital release and utilization of the nutrients by the crops. The application of vermicompost prepared from celotia and Parthenium was also found beneficial in reducing the weed infestation in the groundnut crop. The results of [25] indicated that the Parthenium compost at low amendments with cowdung help its eradication with better utilization. The improved nitrogen, phosphorus, potassium and calcium and decrease in pH and organic carbon was observed by [26] in Parthenium vermicomposted with *Eisenia fetida*. Their results indicated that the Parthenium with cowdung in appropriate quantity can be a raw material for vermicomposting. The vermicompost prepared with Parthenium and cow dung in 3:2 ratio using *Eudrillus eugeneae* and *Eisenia fetida* and found that Parthenium plants without flowers had high pH, N and K than with flowers whereas the phosphorus content was high in parthenium plants composted with flowers [27].

## Conclusions

The adoption of vermicomposting to eradicate the weeds and reduce the problems arising due the weeds in agricultural fields is gaining momentum among farmers. The technology should be utilized effectively in this regard. The present work indicates that the process can be carried out in simple methods and using materials that are available on farm. The weeds can be collected and mixed with organic farm wastes that will add the nitrogen content and increase the nutritive value of the resultant product. The nutrient status of the resultant products promises the utilization of animal wastes and farm wastes based on the availability of farmer’s field for decomposition of Parthenium weed. The vermitechnology in addition to weed reduction and waste utilization also gives an opportunity to entrepreneurship.
